# Supplementary material for: Interactions of outer membrane lipoproteins Pseudomonas aeruginosa PA3214 and Escherichia coli PqiC with their MCE protein binding partners, PA3213 and PqiB
Source: J Biol Chem. 2026 Aug 25;302(10):113486. doi: 10.1016/j.jbc.2026.113486 (PMC13631591; doi:10.1016/j.jbc.2026.113486)
Supplement: Supplementary file 1 [file mmc4.pdf]

Supporting Information

Interactions of outer membrane lipoproteins *Pseudomonas aeruginosa* PA3214 and *Escherichia coli* PqiC with their MCE protein binding partners, PA3213 and PqiB

Sabrina I. Giacometti[1], Nicolas Coudray[1,2], Rachel L. Redler[1], Gira Bhabha[1*] and Damian C. Ekiert[1*]

[1]Department of Biology, Johns Hopkins University, Baltimore, MD, USA
[2]Applied Bioinformatics Laboratories, New York University School of Medicine, New York, USA

*For correspondence: gira.bhabha@be-labs.org and damian.ekiert@be-labs.org

List of Contents Included in this File

Supplementary Figure S1. Examples of MCE systems in *E. coli* and mycobacteria
Supplementary Figure S2. Biochemical and structural characterization of PA3214^{sol}
Supplementary Figure S3. Cryo-EM data processing workflow for the structure of PA3214^{sol}
Supplementary Figure S4. Map validation corresponding to the workflow in Fig. S3 (Map 1)
Supplementary Figure S5. Biochemical and structural characterization of PA3211-PA3214 overexpressed in *E. coli* and purified using His-tagged PA3211
Supplementary Figure S6. Cryo-EM data processing workflow for the PA3214 full length structure in complex with PA3213 C-terminal peptide
Supplementary Figure S7. Map validation corresponding to the workflow in Fig. S6
Supplementary Figure S8. Local refinement on the consensus map to assign the orientation of PA3214 relative to the rest of the complex
Supplementary Figure S9. Results and analysis of PqiC DMS experiments, related to Figures 3-5
Supplementary Figure S10. Pull down assays probing the interaction between co-purified PA3213:PA3214 and PqiB:PqiC
Supplementary Figure S11. Representative cryo-EM map densities
Supplementary Table S1. Cryo-EM data collection for PA3214^{sol}
Supplementary Table S2. Refinement and validation statistics for PA3214^{sol}
Supplementary Table S3. Cryo-EM data collection for PA3213-PA3214 subcomplex
Supplementary Table S4. Refinement and validation statistics for PA3213-PA3214 subcomplex
Supplementary Table S5. Bacterial strains and constructs used in this study

Other Supporting Materials for this Manuscript

Supplementary Movie S1. Result of multi-body refinement in Relion, using the closed conformation (Map 2), showing tilting of the PA3214 lipoprotein relative to the PA3213 MCE needle.
Supplementary Movie S2. Result of multi-body refinement in Relion, using the closed conformation (Map 2), showing rotation of the PA3214 lipoprotein relative to the PA3213 MCE needle.

Supplementary Movie S3. Result of multi-body refinement in Relion, using the closed conformation (Map 2), showing sliding of the PA3214 lipoprotein relative to the PA3213 MCE needle.

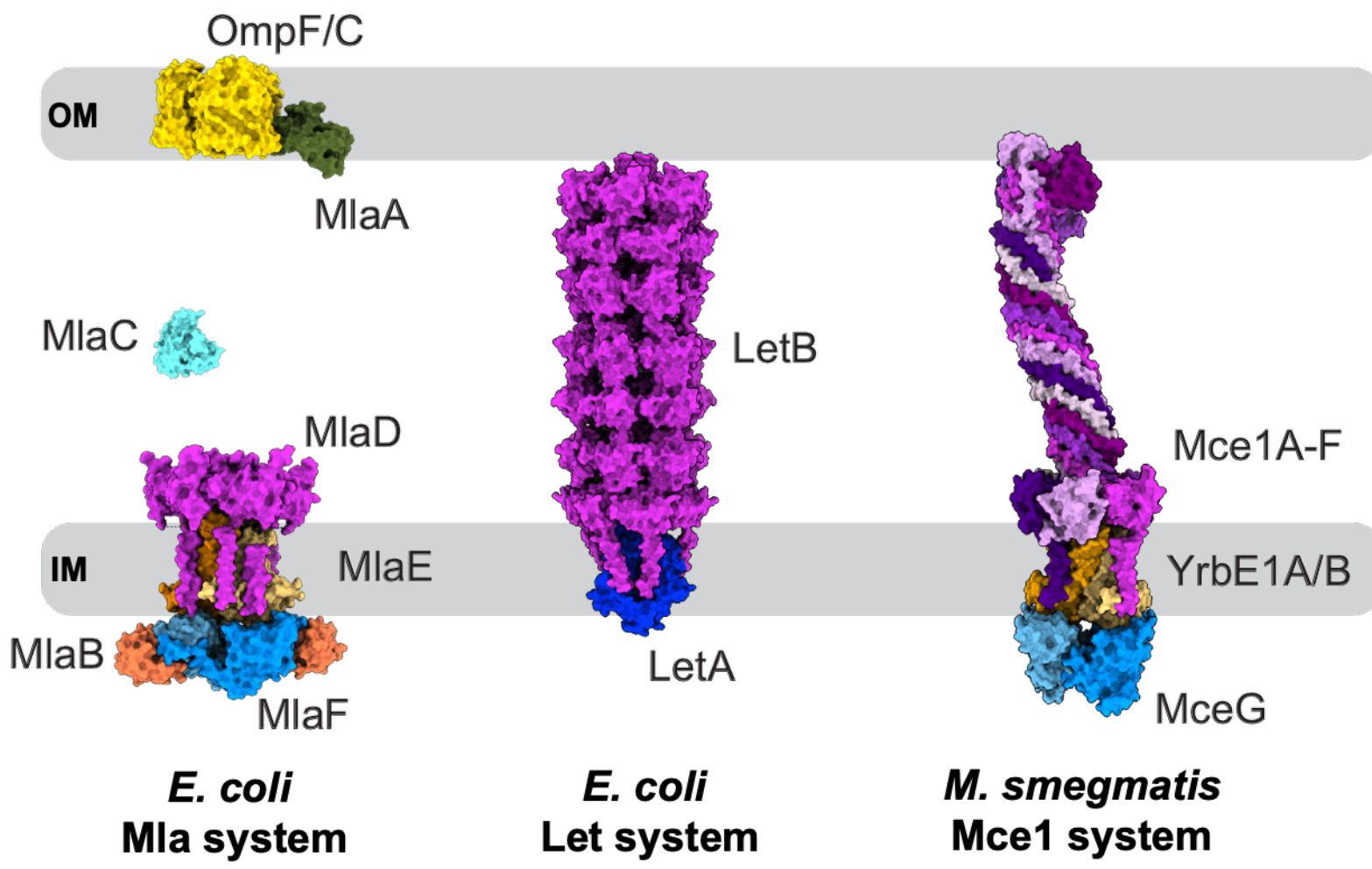

Supplementary Figure S1. Examples of MCE systems in *E. coli* and mycobacteria. *E. coli* Mla system (PDB 6XBD, 5UWA, 5NUR), *E. coli* Let system (PDB 9N8W) and *M. smegmatis* Mce1 (PDB 8FEE) MCE systems in the context of a double-membraned cell envelope. MalD, LetB and Mce1A-F are the MCE proteins in these systems.

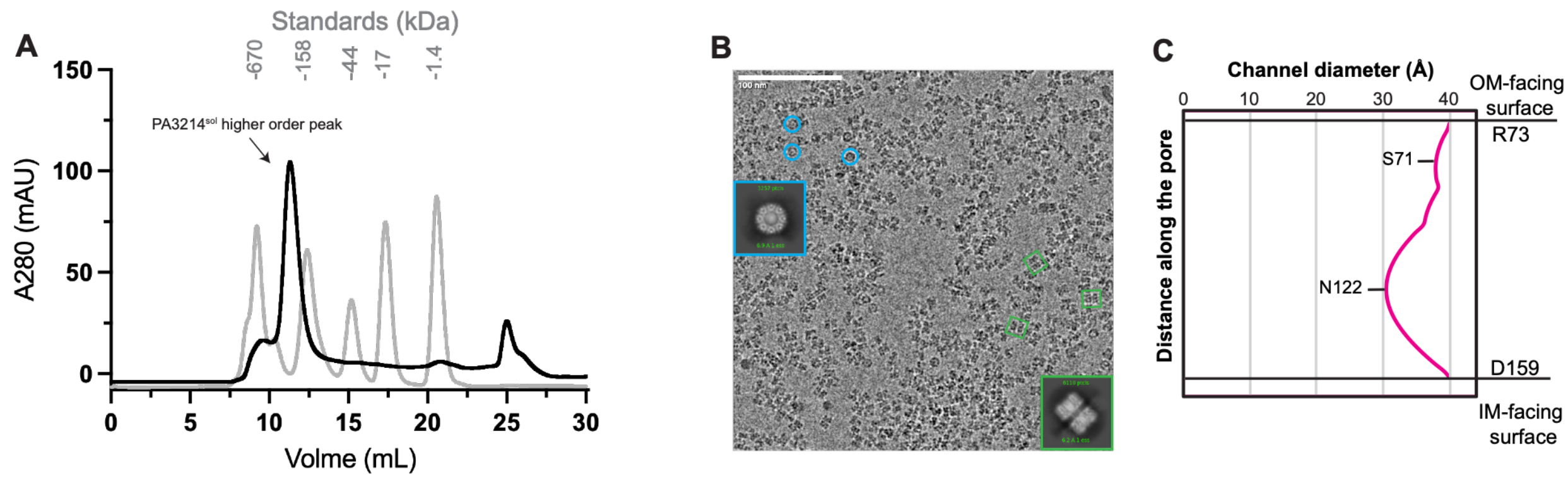

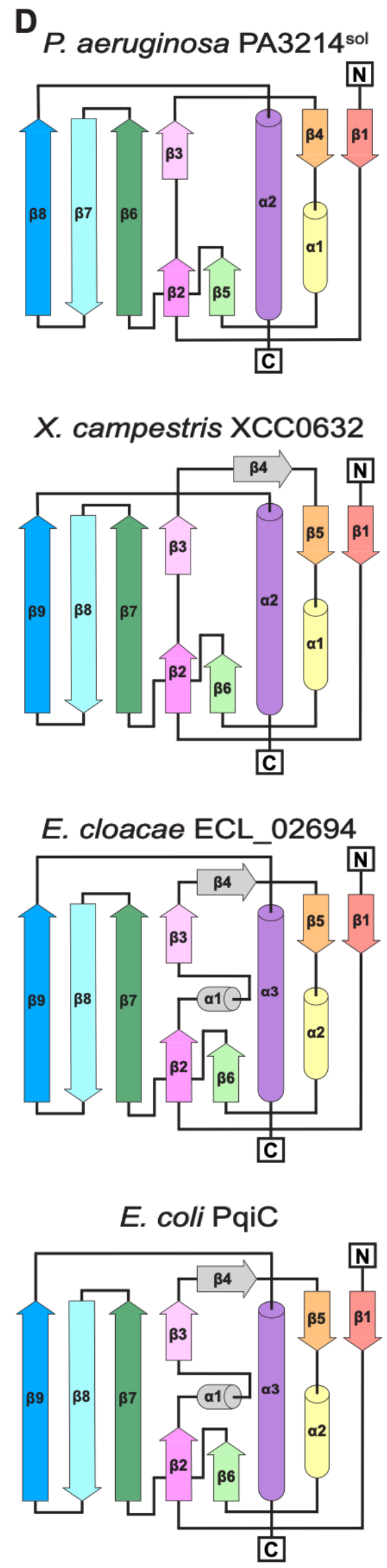

E

Structural Similarity (E-value)

	P. aeruginosa PA3214	*X. campestris* XCC0632	*E. cloacae* ECL_02694	*E. coli* PqiC
X. campestris XCC0632	3.31 x 10^{-18}	8.58 x 10^{-35}	6.77 x 10^{-10}	1.92 x 10^{-9}
E. cloacae ECL_02694	2.6 x 10^{-10}	1.17 x 10^{-8}	4.57 x 10^{-36}	2.36 x 10^{-28}
E. coli PqiC	1.8 x 10^{-10}	7.16 x 10^{-9}	1.12 x 10^{-28}	1.64 x 10^{-37}

Sequence Identity

	P. aeruginosa PA3214	*X. campestris* XCC0632	*E. cloacae* ECL_02694	*E. coli* PqiC
P. aeruginosa PA3214	100%	33.5%	13.5%	12.6%
X. campestris XCC0632	33.5%	100%	16.4%	13.2%
E. cloacae ECL_02694	13.5%	15.7%	100%	78.8%
E. coli PqiC	12.6%	13.8%	78.8%	100%

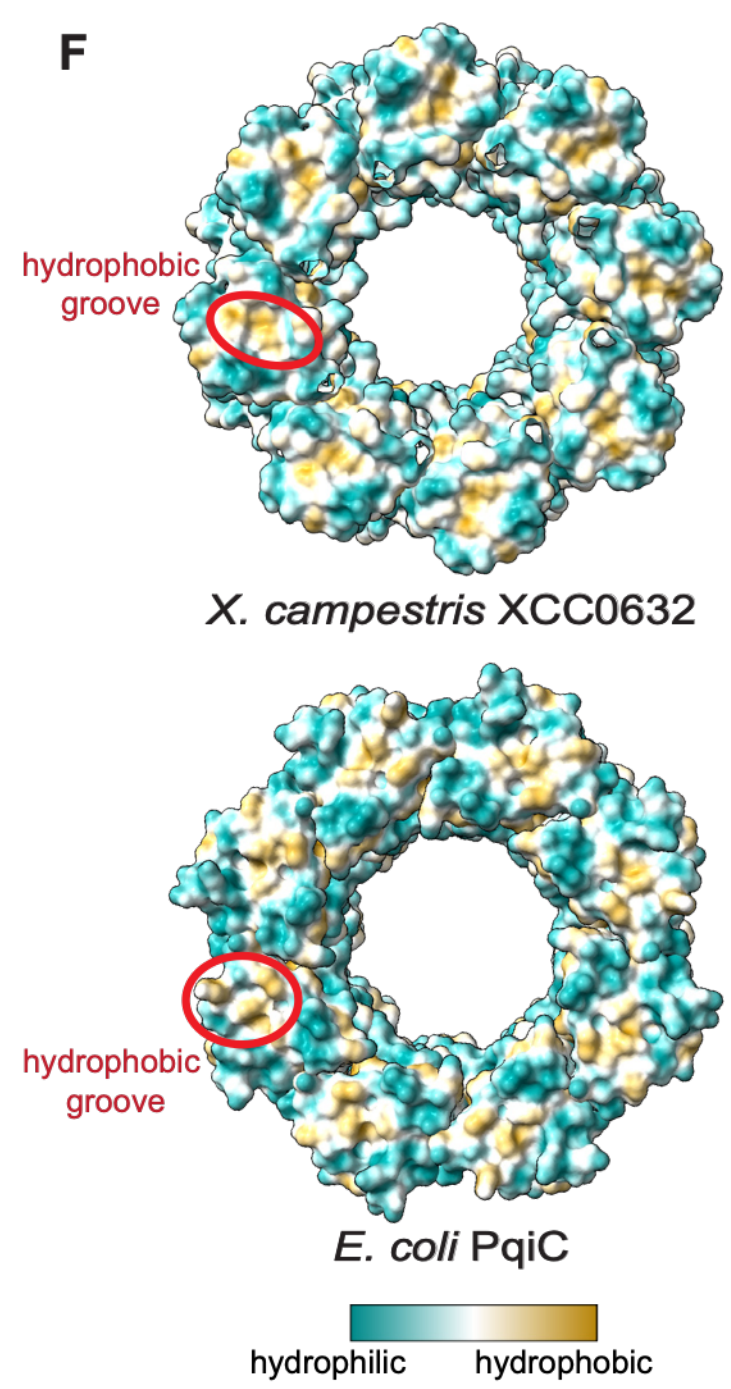

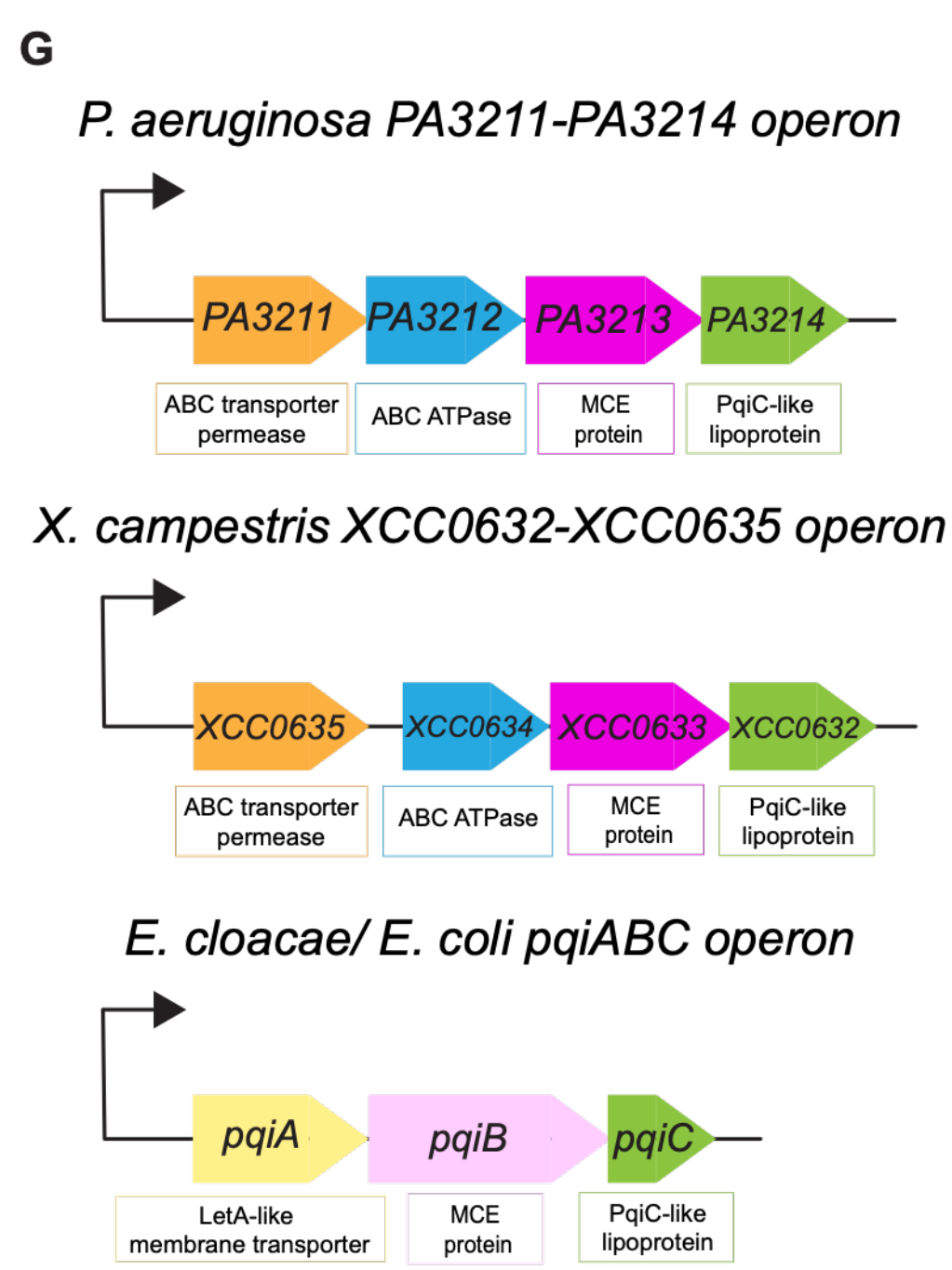

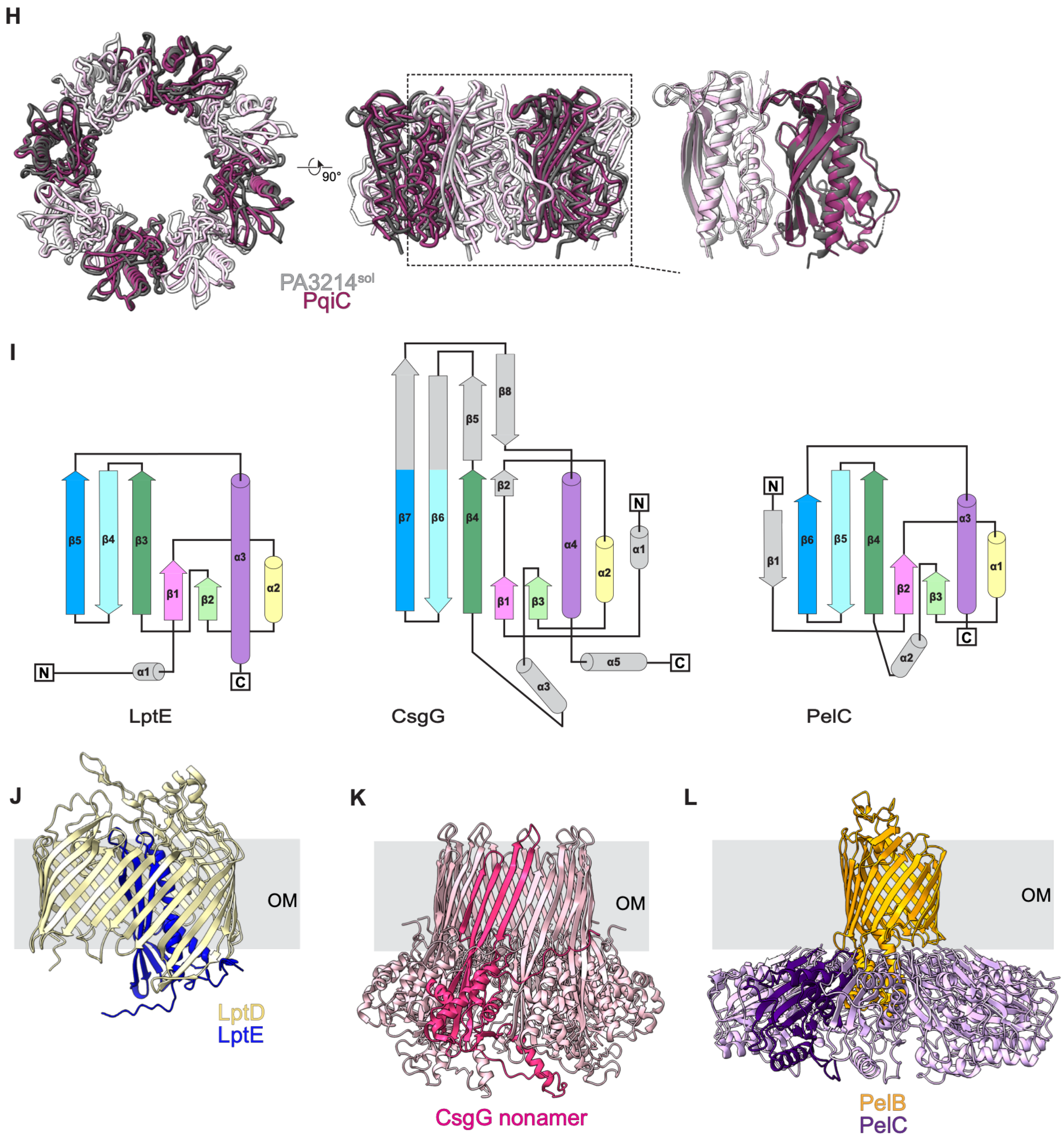

Supplementary Figure S2. Biochemical and structural characterization of PA3214^{sol}. *A,* a representative size exclusion chromatogram for PA3214^{sol} (black) overlaid with standards (gray). The PA3214^{sol} higher order complex elutes between the 158 and 670 kDa protein standard. *B,* example of a typical PA3214^{sol} cryo-EM micrograph. Examples of top views are indicated with blue circles and the blue inset shows a representative 2D class. Examples of side views are indicated with green rectangles and the green inset shows a representative 2D class, highlighting the non-specific interaction between two rings. *C,* diameter of the PA3214^{sol} pore (magenta) along the length of the lipoprotein from the OM-facing surface to the IM-facing surface, measured using CHAP (84). *D,* topology diagrams of *P. aeruginosa* PA3214^{sol}, *X. campestris* XCC0632 (PDB 2IQI), *E. cloacae* PqiC (PDB 6OSX), and *E. coli* PqiC (PDB 8Q2C) highlighting structural homology compared to PA3214^{sol} (colored elements) and features not found in PA3214 in gray. *E,* comparison of structural similarity and sequence identity between *P. aeruginosa* PA3214^{sol}, *X. campestris* XCC0632, *E. cloacae* PqiC and *E. coli* PqiC. Values obtained from FoldSeek search

(44). *F,* molecular surface representation of the OM-facing surfaces of *X. campestris* XCC0632 (PDB 2IQI) and *E. coli* PqiC (PDB 8Q2C) colored by hydrophobicity. Hydrophobic groove indicated by red circle. *G,* schematic of *P. aeruginosa PA3211-PA3214*, *X. campestris XCC0632-XCC0635*, and *E. cloacae* / *E. coli pqiABC* operons. *H,* Overlay of PA3214^{sol} and PqiC, aligned on the octamer. *I,* topology diagrams of LptE (PDB 4N4R), CsgG (PDB 7BRM) and PelC (5T10) highlighting structural homology compared to PA3214 (colored elements) and features not found in PA3214 in gray. *J,* cartoon representation of the LptDE complex (PDB 4N4R) relative to the OM (gray). *K,* cartoon representation of CsgG nonamer (PDB 7BRM), with a monomer highlighted in hot pink, relative to the OM (gray). *L,* a cartoon representation of the PelBC complex (PDB 9H80), with a monomer of PelC highlighted in dark purple, relative to the OM (gray).

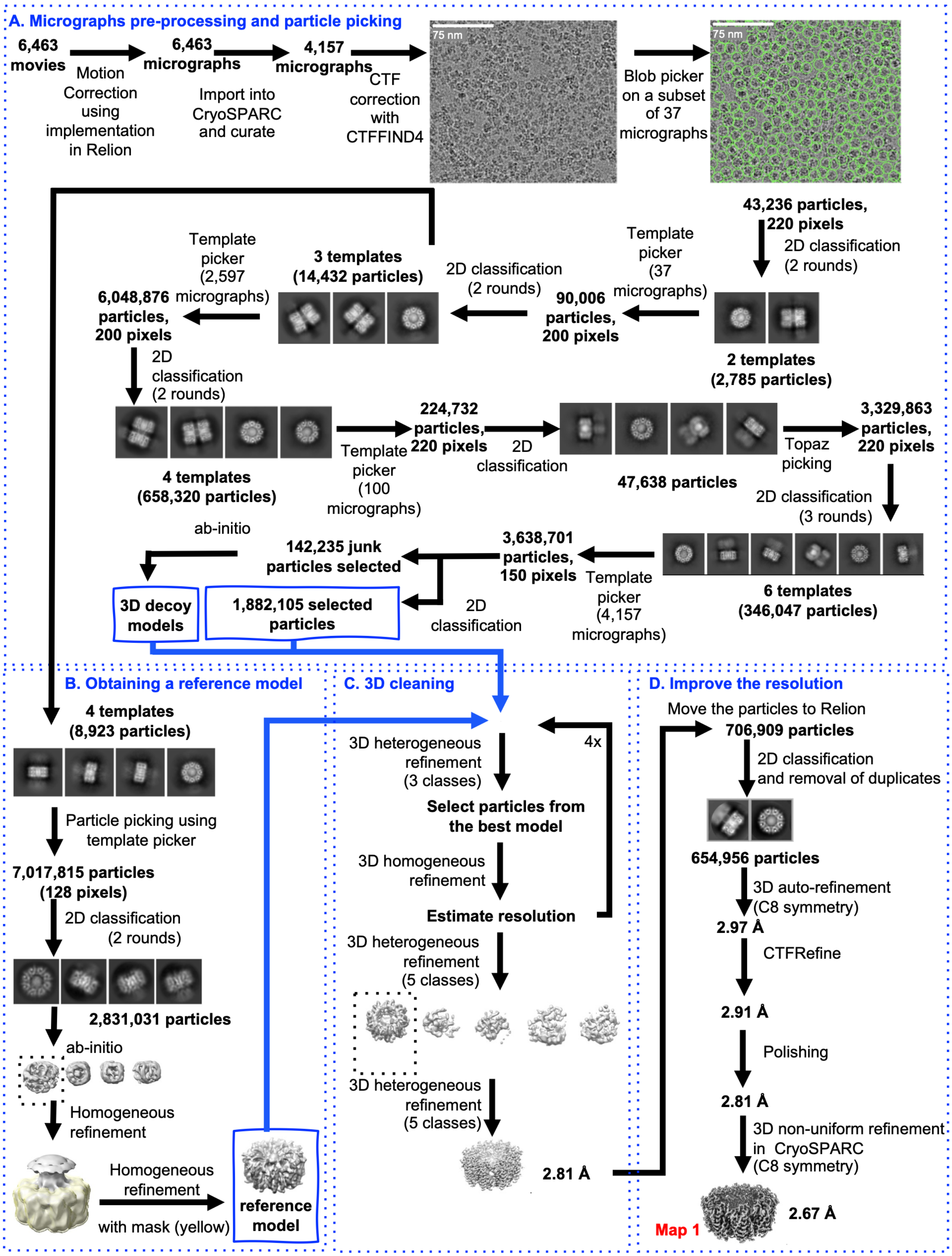

Supplementary Figure S3. Cryo-EM data processing workflow for the structure of PA3214^{sol}. *A*, micrographs pre-processing and particle picking. *B*, Generating a reference model

of a single PA3214$_{sol}$ ring. *C*, 3D cleaning of the dataset. *D*, steps implemented to improve the resolution, leading to the final model. See Methods for details.

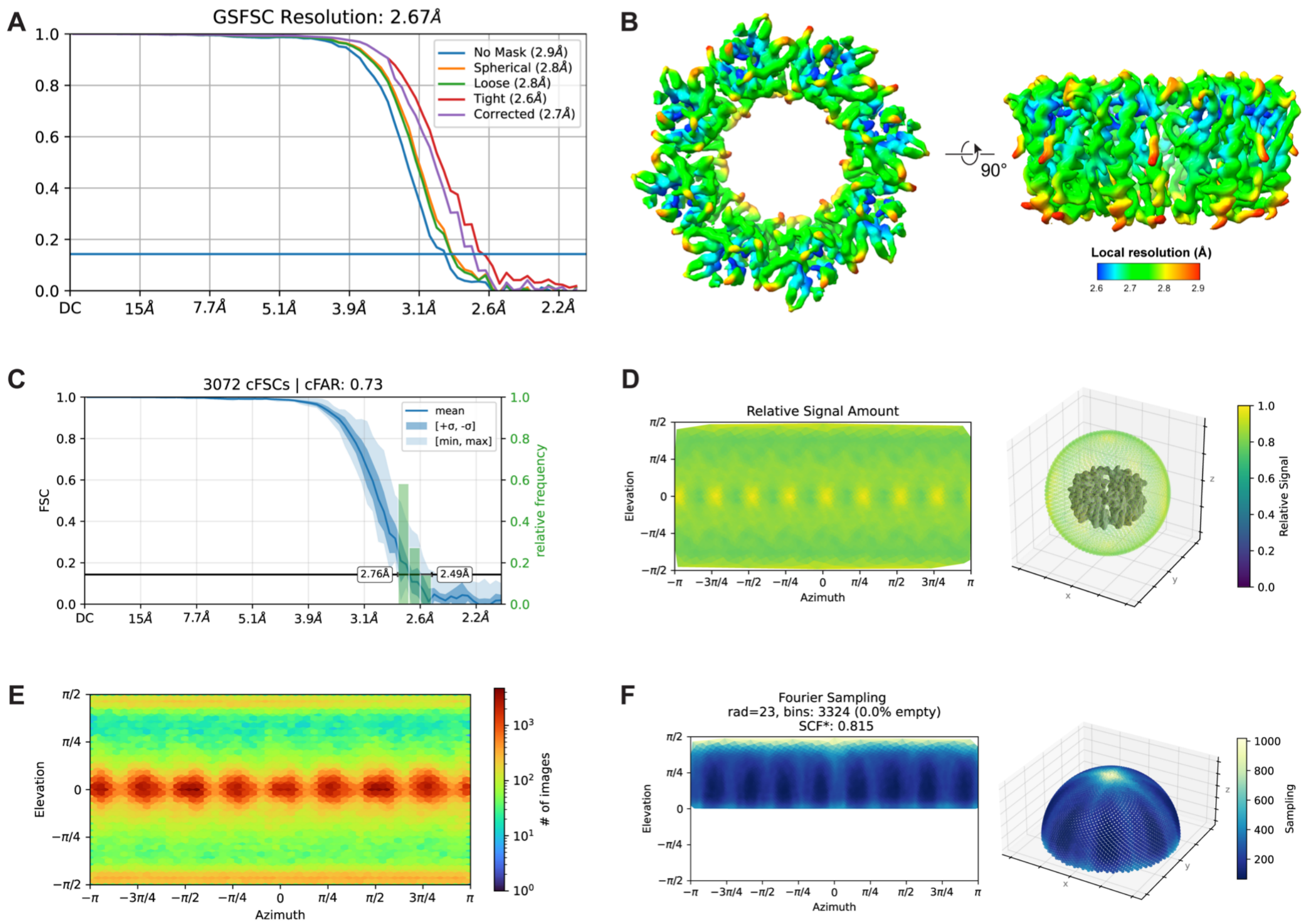

Supplementary Figure S4. Map validation corresponding to the workflow in Fig. S3 (Map 1). *A*, overall Gold Standard Fourier Shell Coefficient (GSFSC) curve. *B*, local resolution of PA3214^{sol} map, shown in C8, unfiltered, at a threshold of 0.3. *C*, canonical FSC (cFSC) curve. *D*, relative signal amount by viewing direction plots. *E*, viewing direction distribution. *F*, fourier sampling plot . All plots were generated with CryoSPARC.

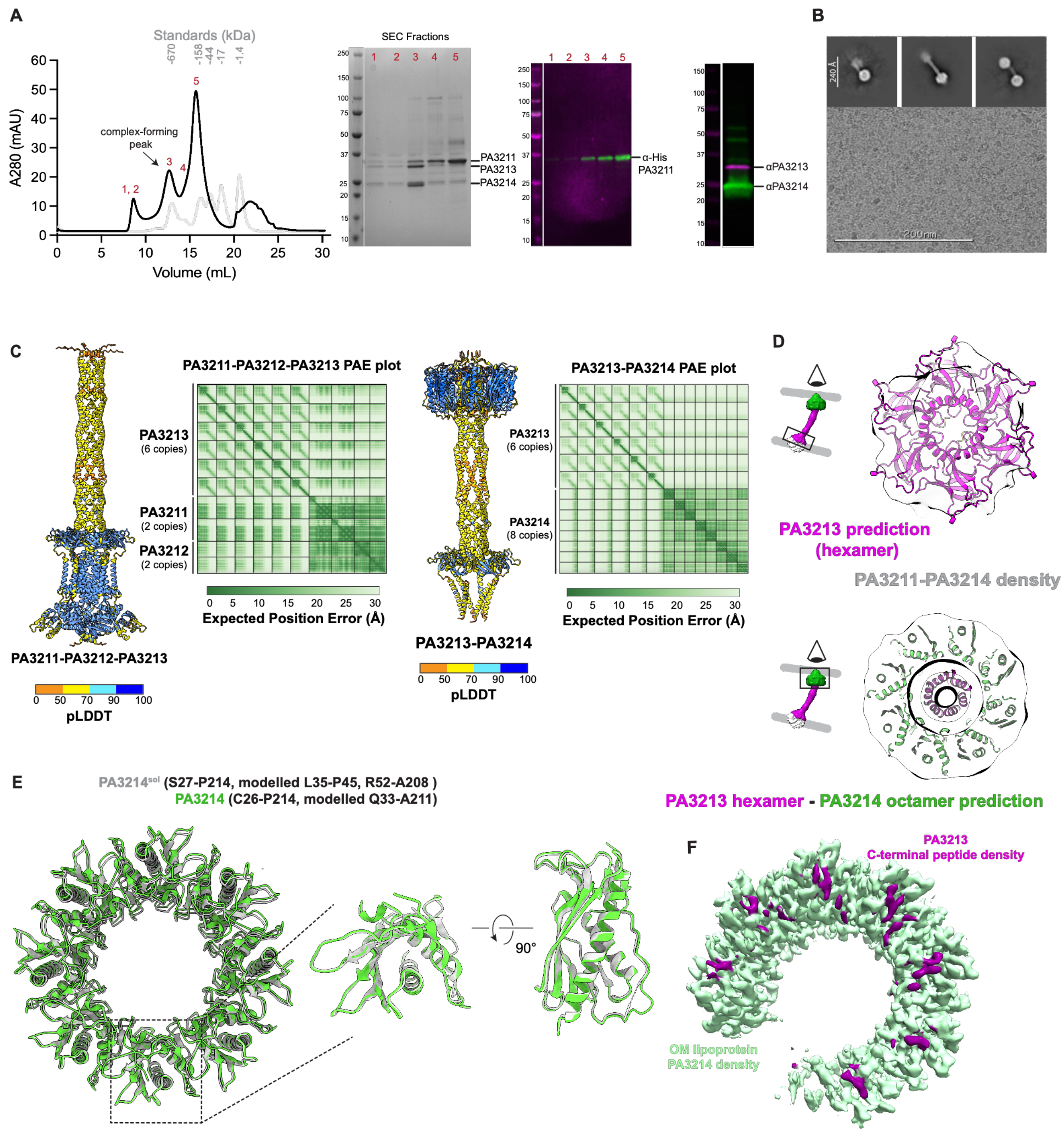

Figure S5. Biochemical and structural characterization of PA3211-PA3214 overexpressed in *E. coli* and purified using His-tagged PA3211. *A,* representative size exclusion chromatogram for PA3211-PA3214 complex (black) overlaid with standards (gray) and 5 peak fractions analyzed shown in red. Corresponding SDS-PAGE stained with Coomassie, and Western blot are shown. On the right, a representative anti-PA3213 and anti-PA3214 Western blot of the same plasmid. *B,* representative cryo-EM micrograph for PA3211-PA3214 sample, with an inset showing 2D classes. *C,* AlphaFold 3 predictions of PA3211-PA3212-PA3213 and PA3213-PA3214 that were used to prepare a composite prediction of the PA3211-PA3214 complex in Fig. 2C. The stoichiometry used in the predicted complex (PA3211:PA3212:PA3213:PA3214 2:2:6:8) is based on previously determined structures of homologous proteins and experimental data from this work. Confidence statistics for each

prediction are shown with pLDDT (Predicted Local Distance Difference Test) values colored as in the corresponding key; predicted aligned error (PAE) values between the proteins of each prediction plotted on the corresponding heatmap. *D,* top view of PA3211-PA3214 complex prediction from AlphaFold showing the MCE hexameric ring assembly (magenta), or the PA3214 octamer assembly (green) docked into the low resolution consensus map (Map 4: C1 symmetry, unfiltered, at threshold 0.126). *E,* overlay of the cartoon representation of PA3214^{sol} structure (gray) with the full-length PA3214 structure in the closed conformation (green). *F*, density for PA3214 in the open conformation (green) and the PA3213 C-terminal peptide (magenta) (Map 3: C1 symmetry, sharpened, with a threshold of 0.4728, and displayed to show only the densities around PA3214 using the “volume zone #1 near sel range 5" command in ChimeraX, where #1 is the map and sel is the model).

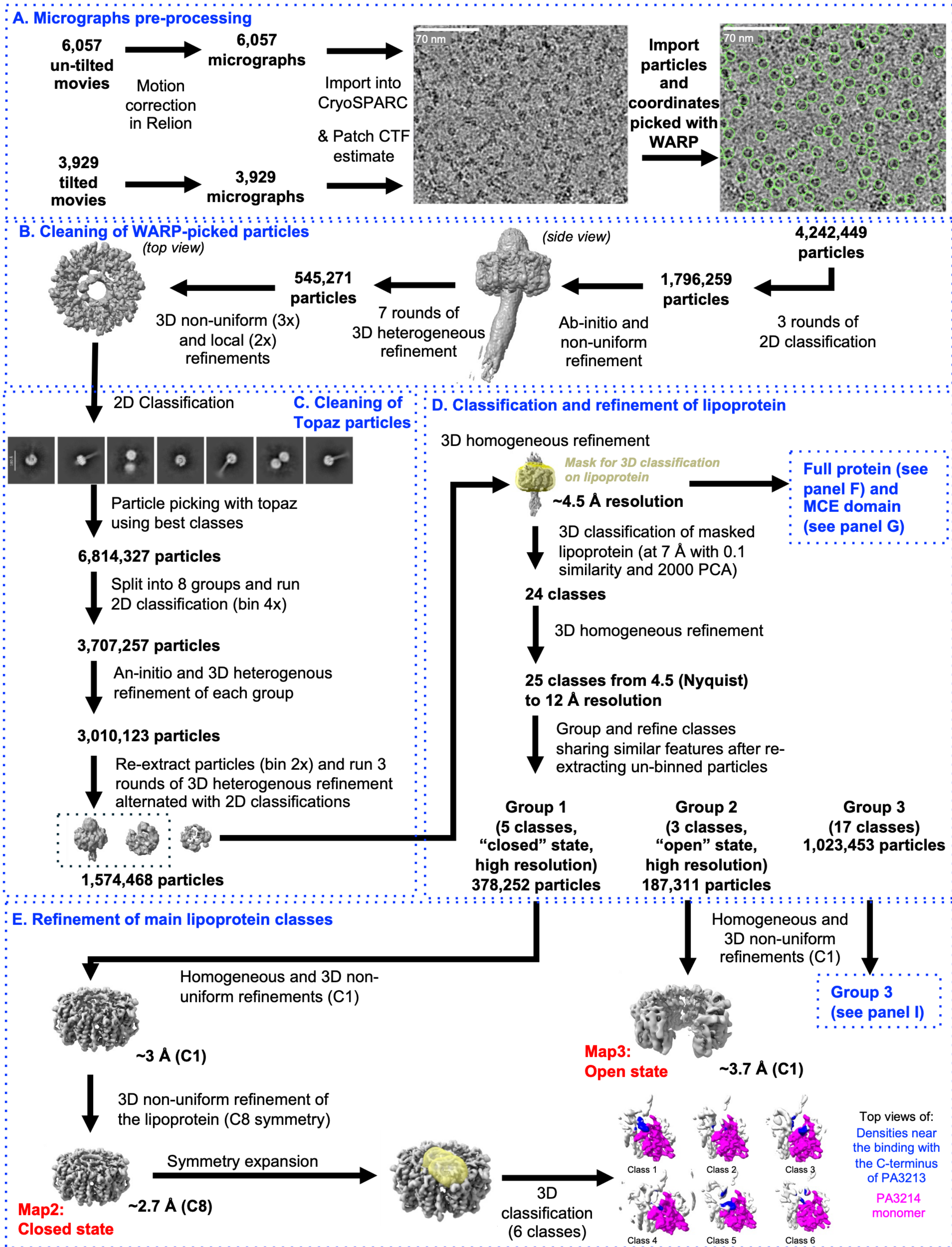
A. Micrographs pre-processing
6,057 un-tilted movies
6,057 micrographs
Motion correction in Relion
Import into CryoSPARC
& Patch CTF estimate
3,929 tilted movies
3,929 micrographs
70 nm
Import particles and coordinates picked with WARP
B. Cleaning of WARP-picked particles
(top view)
(side view)
4,242,449 particles
3 rounds of 2D classification
1,796,259 particles
Ab-initio and non-uniform refinement
7 rounds of 3D heterogeneous refinement
545,271 particles
3D non-uniform (3x) and local (2x) refinements
2D Classification
C. Cleaning of Topaz particles
Particle picking with topaz using best classes
6,814,327 particles
Split into 8 groups and run 2D classification (bin 4x)
3,707,257 particles
An-initio and 3D heterogenous refinement of each group
3,010,123 particles
Re-extract particles (bin 2x) and run 3 rounds of 3D heterogenous refinement alternated with 2D classifications
1,574,468 particles
D. Classification and refinement of lipoprotein
3D homogeneous refinement
Mask for 3D classification on lipoprotein
~4.5 Å resolution
Full protein (see panel F) and MCE domain (see panel G)
3D classification of masked lipoprotein (at 7 Å with 0.1 similarity and 2000 PCA)
24 classes
3D homogeneous refinement
25 classes from 4.5 (Nyquist) to 12 Å resolution
Group and refine classes sharing similar features after re-extracting un-binned particles
Group 1 (5 classes, “closed” state, high resolution) 378,252 particles
Group 2 (3 classes, “open” state, high resolution) 187,311 particles
Group 3 (17 classes) 1,023,453 particles
E. Refinement of main lipoprotein classes
Homogeneous and 3D non-uniform refinements (C1)
~3 Å (C1)
3D non-uniform refinement of the lipoprotein (C8 symmetry)
Map2: Closed state
~2.7 Å (C8)
Symmetry expansion
3D classification (6 classes)
Homogeneous and 3D non-uniform refinements (C1)
Group 3 (see panel I)
Map3: Open state
~3.7 Å (C1)
Class 1
Class 2
Class 3
Class 4
Class 5
Class 6
Top views of:
Densities near the binding with the C-terminus of PA3213
PA3214 monomer

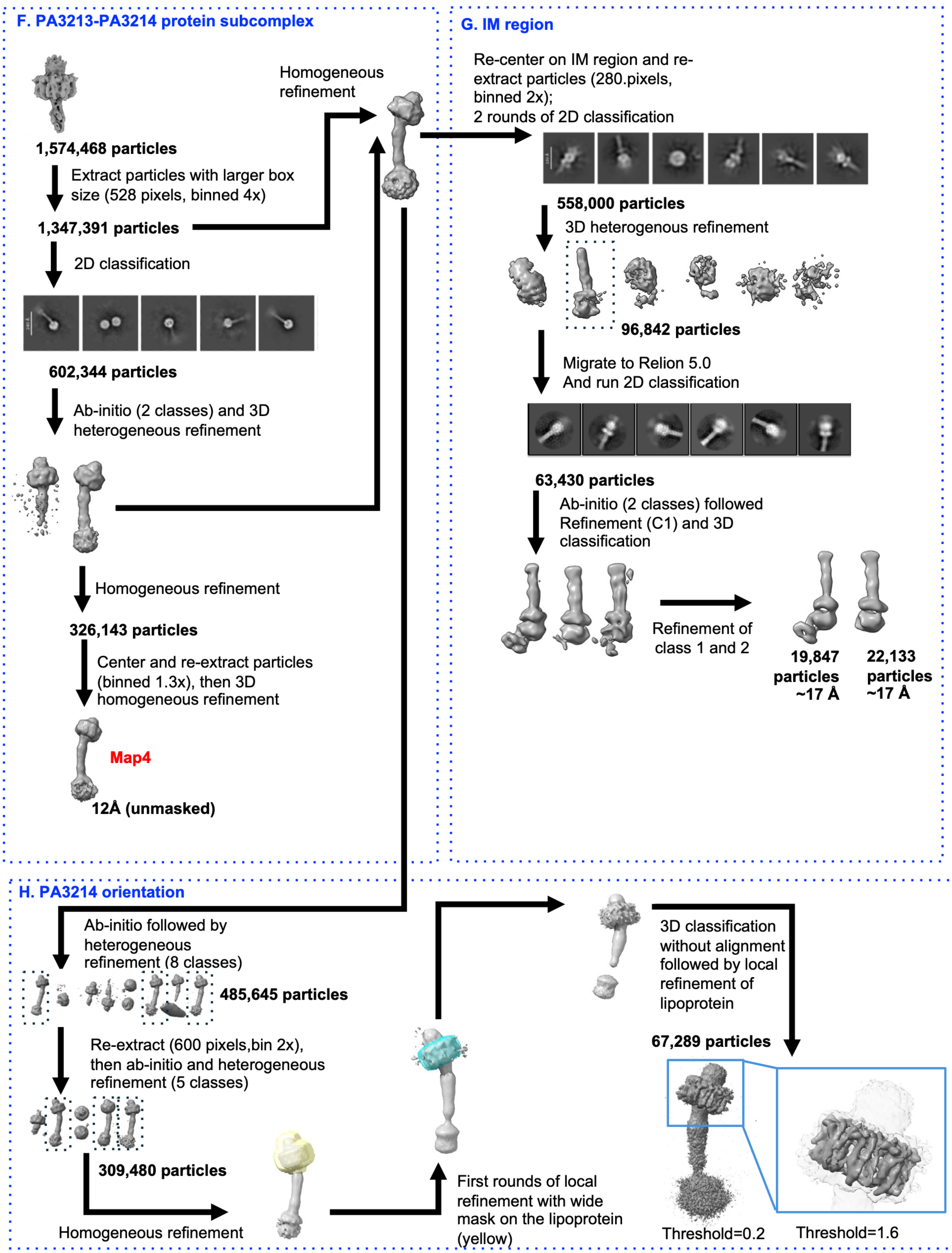
F. PA3213-PA3214 protein subcomplex
1,574,468 particles
Extract particles with larger box size (528 pixels, binned 4x)
1,347,391 particles
Homogeneous refinement
2D classification
602,344 particles
Ab-initio (2 classes) and 3D heterogeneous refinement
Homogeneous refinement
326,143 particles
Center and re-extract particles (binned 1.3x), then 3D homogeneous refinement
Map4
12Å (unmasked)
G. IM region
Re-center on IM region and re-extract particles (280.pixels, binned 2x);
2 rounds of 2D classification
558,000 particles
3D heterogenous refinement
96,842 particles
Migrate to Relion 5.0
And run 2D classification
63,430 particles
Ab-initio (2 classes) followed Refinement (C1) and 3D classification
Refinement of class 1 and 2
19,847 particles ~17 Å
22,133 particles ~17 Å
H. PA3214 orientation
Ab-initio followed by heterogeneous refinement (8 classes)
485,645 particles
Re-extract (600 pixels,bin 2x), then ab-initio and heterogeneous refinement (5 classes)
309,480 particles
Homogeneous refinement
First rounds of local refinement with wide mask on the lipoprotein (yellow)
3D classification without alignment followed by local refinement of lipoprotein
67,289 particles
Threshold=0.2
Threshold=1.6

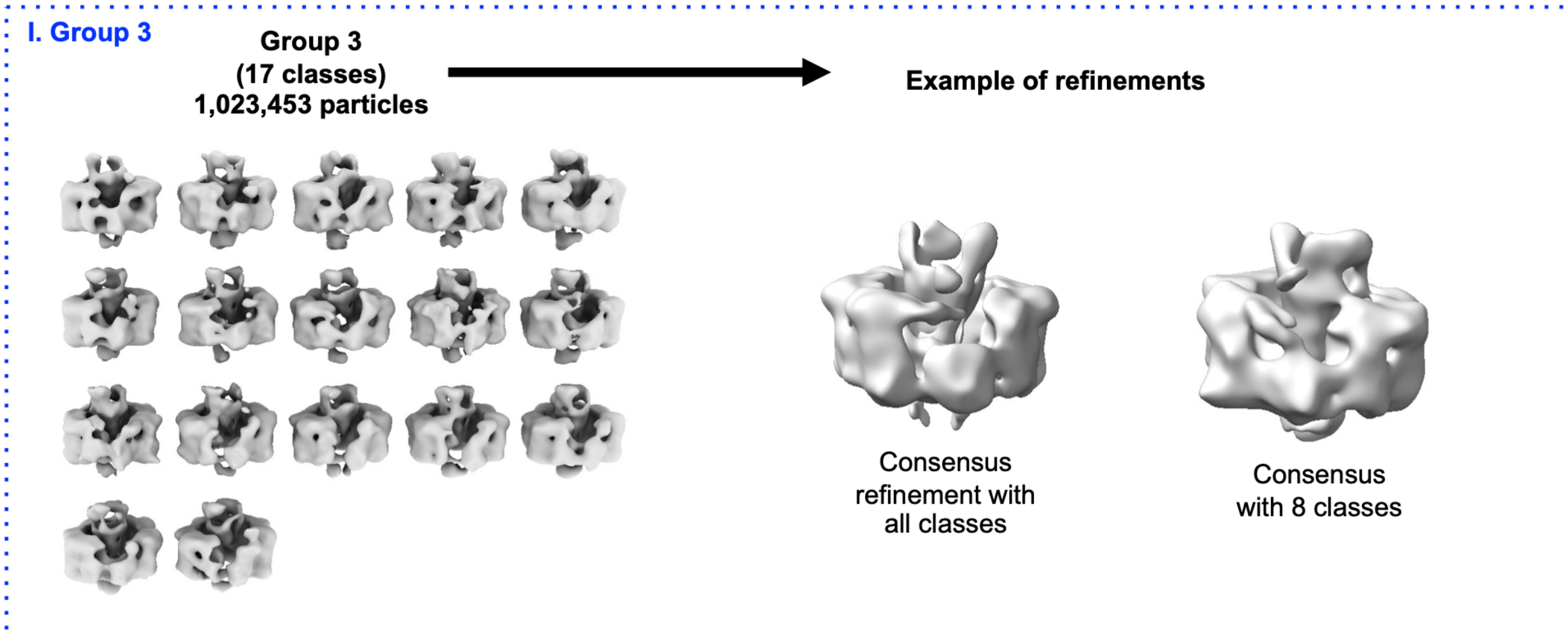

Supplementary Figure S6. Cryo-EM data processing workflow for the PA3214 full length structure in complex with PA3213 C-terminal peptide. *A,* micrographs pre-processing and particle picking. *B,* initial round of cleaning WARP-picked particles that led to the first model. *C,* picking with Topaz and sequential cleaning steps. *D,* classification and refinement of the PA3214 lipoprotein. *E,* refinement and classification of particles leading to full-length PA3214 in closed and in open conformations, as well as further classification leading to various occupancy states of the PA3213 peptide in the PA3214 groove. *F,* refinement of the PA3213-PA3214 subcomplex. *G,* refinement of the IM region of the complex. *H,* focused refinement of PA3214 from the consensus map to confirm its orientation relative to the rest of the complex. *I,* 3D reconstructions from particles not included in Map 1 (closed conformation) or Map 2 (open conformation).

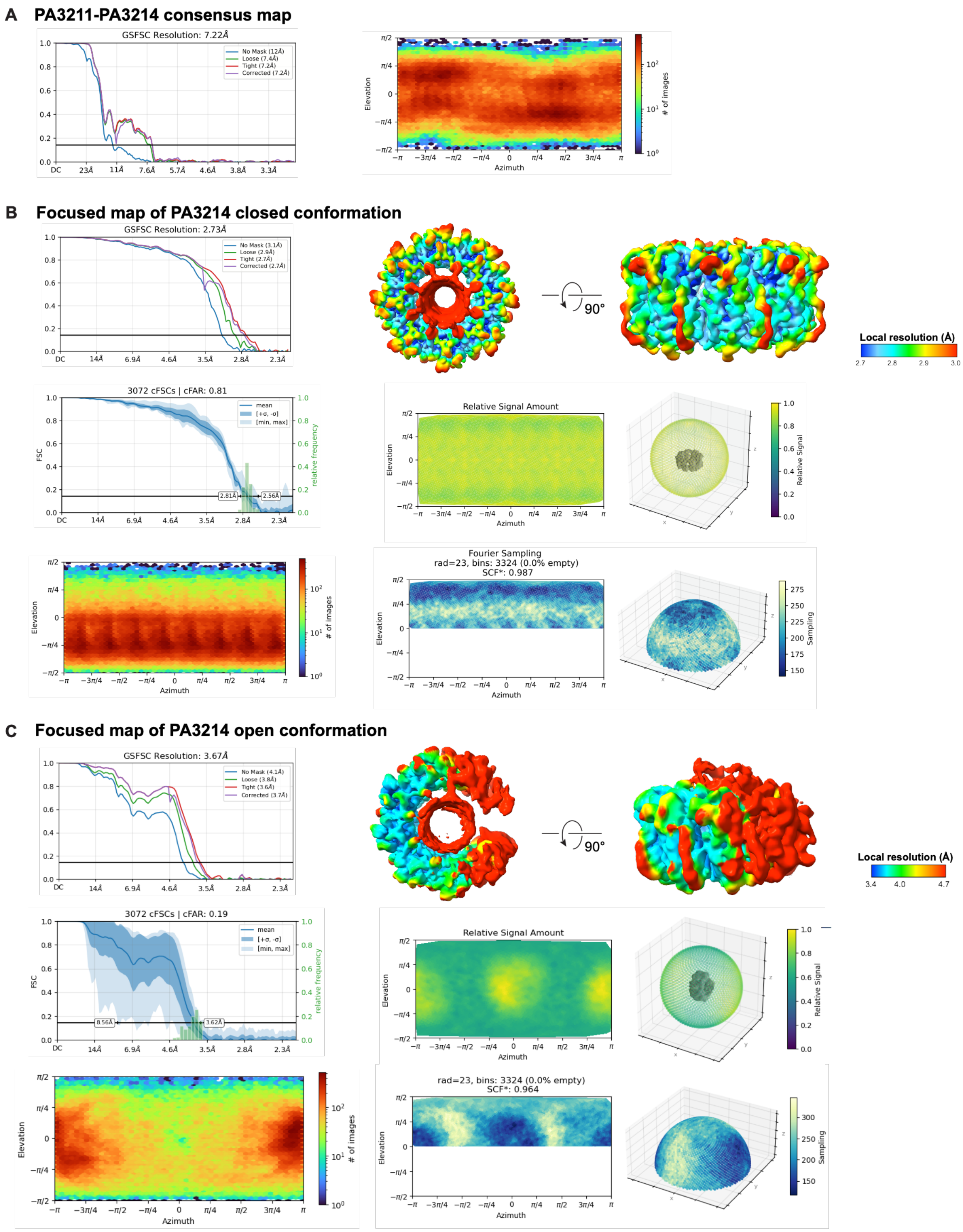

Supplementary Figure S7. Map validation corresponding to the workflow in Fig. S6. *A,* overall resolution and viewing direction distribution of particles in the consensus map (Map 4). *B,* analysis of the full-length PA3214 map in the closed conformation (Map 2: C8 symmetry, unfiltered, at threshold 0.255) with the final overall and local resolution (top row), and orientation diagnostics with the cFSC curve, the canonical FSC summary plot, the relative signal amount vs viewing direction (middle row), the viewing direction distribution, and the Fourier sampling plot (bottom row). *C,* analysis of the full-length PA3214 map in the open conformation (Map 3: C1

symmetry, unfiltered, at threshold 0.246) with the final overall and local resolution (top row), and orientation diagnostics with the cFSC curve, the canonical FSC summary plot, the relative signal amount vs viewing direction (middle row), the viewing direction distribution, and the Fourier sampling plot (bottom row).

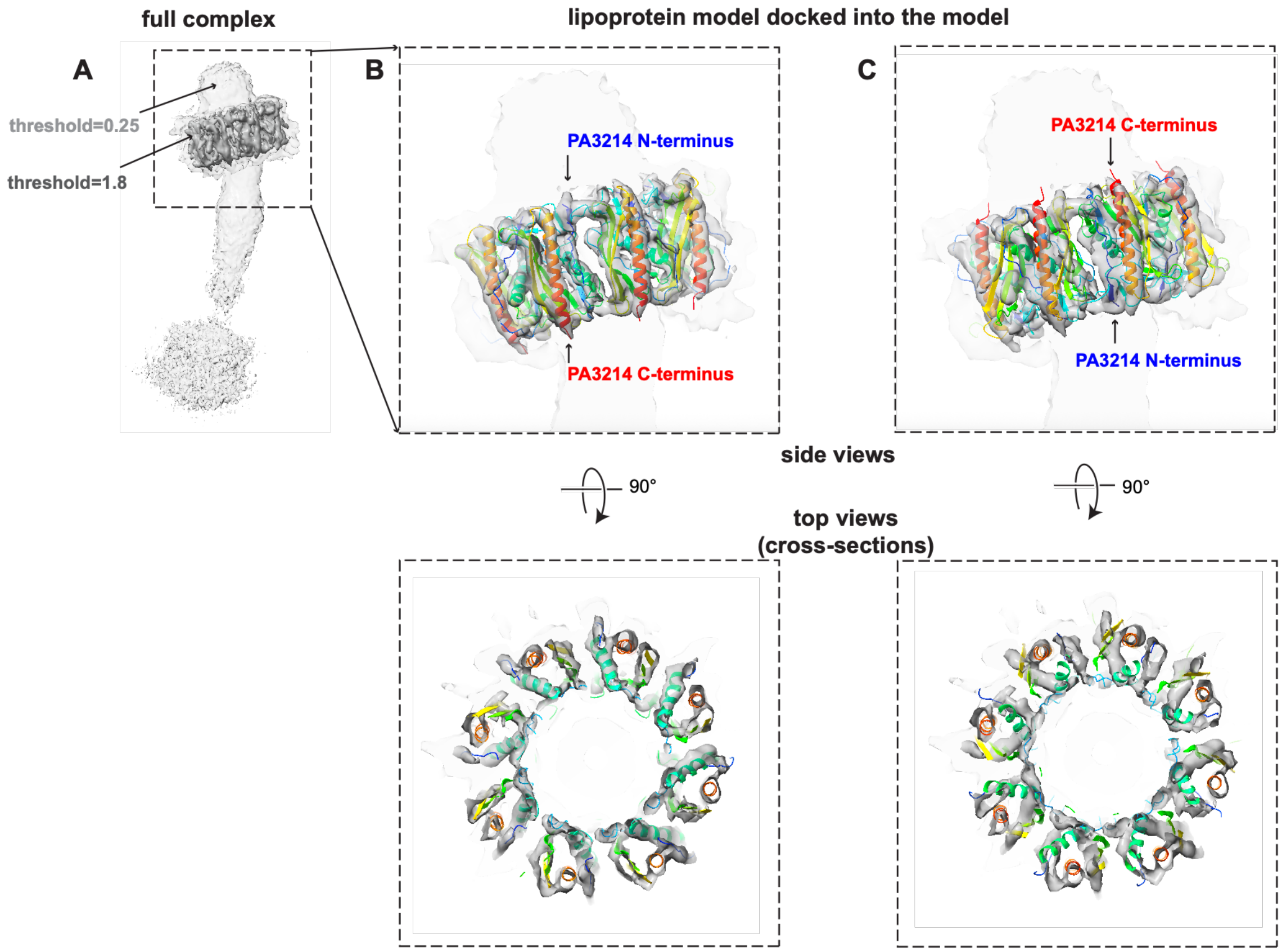

Supplementary Figure S8. Local refinement on the consensus map to assign the orientation of PA3214 relative to the rest of the complex. *A*, locally refined map shown with 2 thresholds, showing the high resolution features of PA3214 as well as the relative position of the PA3211-PA3212-PA3213 domains. *B*, side and top views of the PA3214^{sol} model docked into the map with the PA3214 C-termini facing the OM, which accounts for most of the density. *C,* side and top views of the PA3214^{sol} model docked into the map with the PA3214 C-termini facing the periplasm, which does not fit well into the density, with regions of the model unaccounted for by density, and other regions with empty density.

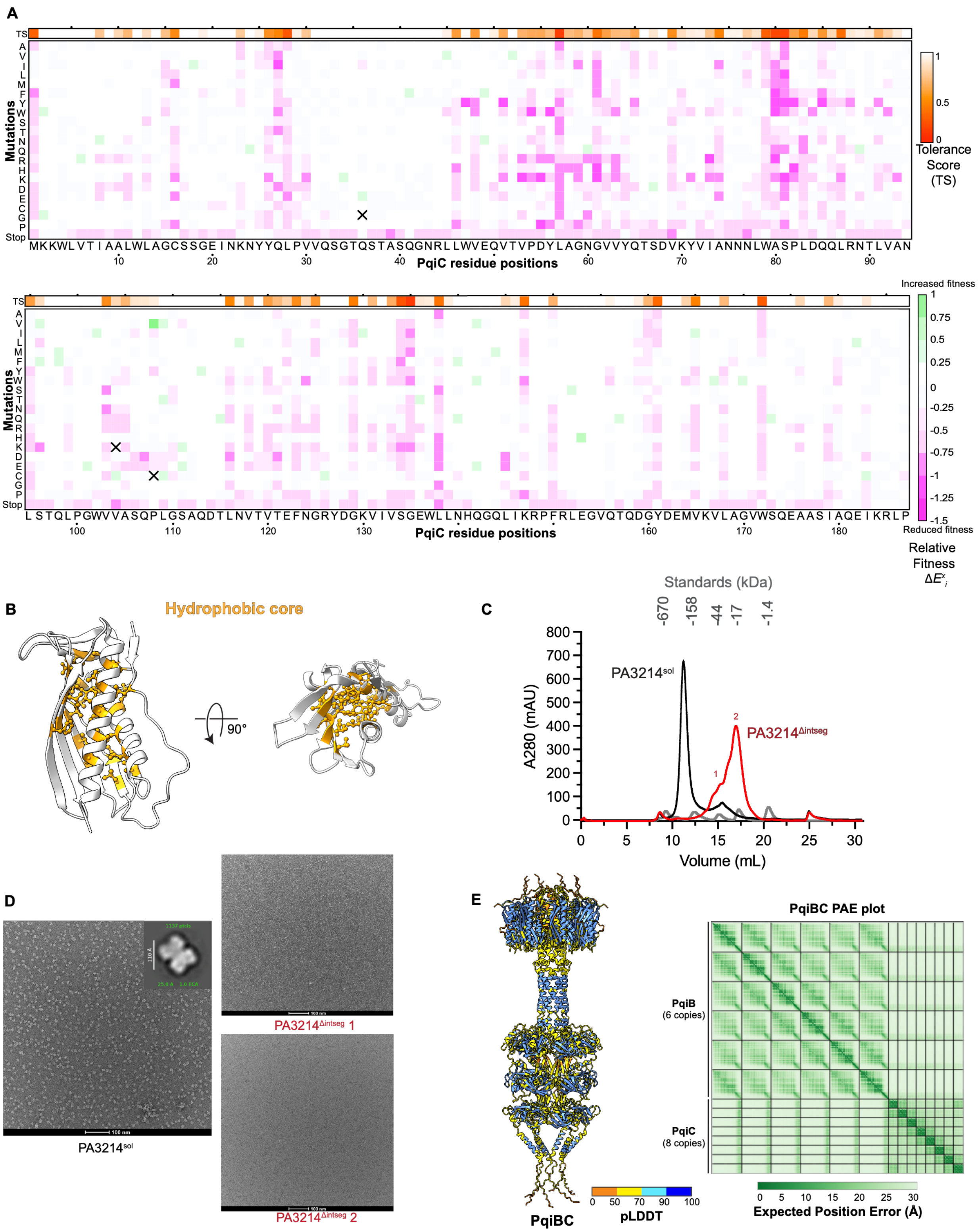
A
TS
Mutations
PqiC residue positions
Tolerance Score (TS)
Increased fitness
Reduced fitness
Relative Fitness ΔE^x_i
B
Hydrophobic core
90°
C
Standards (kDa)
-670 -158 -44 -17 -1.4
PA3214sol
PA3214Δintseg
A280 (mAU)
Volume (mL)
D
PA3214sol
PA3214Δintseg 1
PA3214Δintseg 2
E
PqiBC
pLDDT
PqiBC PAE plot
PqiB (6 copies)
PqiC (8 copies)
Expected Position Error (Å)

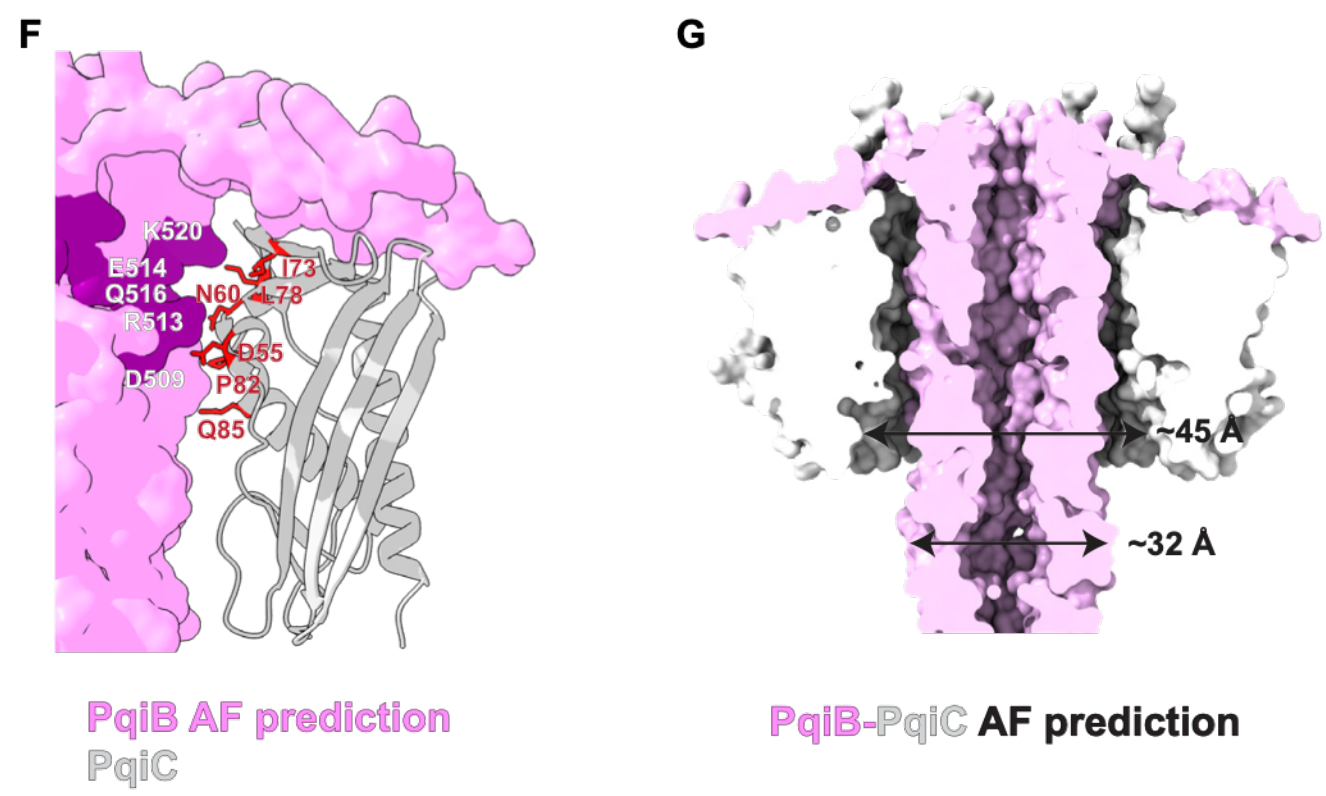

Figure S9. Results and analysis of PqiC DMS experiments, related to Figures 3-5. *A,* heat map summarizing the results of PqiC deep mutational scanning (DMS). Each square represents the average fitness cost (over two biological replicates) of an individual mutation relative to the WT sequence. Mutations that are more harmful to fitness relative to the WT are shown in shades of magenta, while mutations that are more beneficial to fitness are in shades of green, and white represents neutral mutations, as shown in the key. The Tolerance Score (calculated as described in the Methods) is shown as the colored square above each strip. Squares that contain an "X" indicate incomplete coverage at that position. *B,* PqiC monomer (PDB 8Q2C) highlighting the positions of residues with tolerance score <0.9 within the hydrophobic core of the protein (orange). *C,* size exclusion chromatogram for PA3214^{sol} (black) overlaid with PA3214$^{\Delta intseg}$ (red) and standards (gray), with two peaks of PA3214$^{\Delta intseg}$ chosen for analysis by negative staining shown in red numbers. PA3214$^{\Delta intseg}$ elutes between the 44 and 17 kDa protein standards. *D,* representative negative stain micrographs from PA3214^{sol} and PA3214$^{\Delta intseg}$, with an inset of PA3214^{sol} 2D class average, showing oligomers as expected. Neither peak for PA3214$^{\Delta intseg}$ showed clear oligomeric assembly. As PA3214 monomers are only ~20 kDa, we do not expect to resolve these clearly by negative staining. *E,* AlphaFold 3 prediction of PqiBC colored by pLDDT as in the corresponding key, and plot of predicted alignment error (PAE) plotted as a heatmap. *F,* Alphafold 3 PqiB prediction (pink) docked on PqiC (PDB 82QC) (white). The AlphaFold 3 model of the PqiBC complex was predicted, and aligned on the experimental coordinates of PqiC (PDB 82QC) to generate this figure. Potential interactions occurring within the PqiC pore are mapped. *G,* surface representation of PqiBC prediction used in *E*. Black arrows represent the predicted width of the PqiB needle and the PqiC pore.

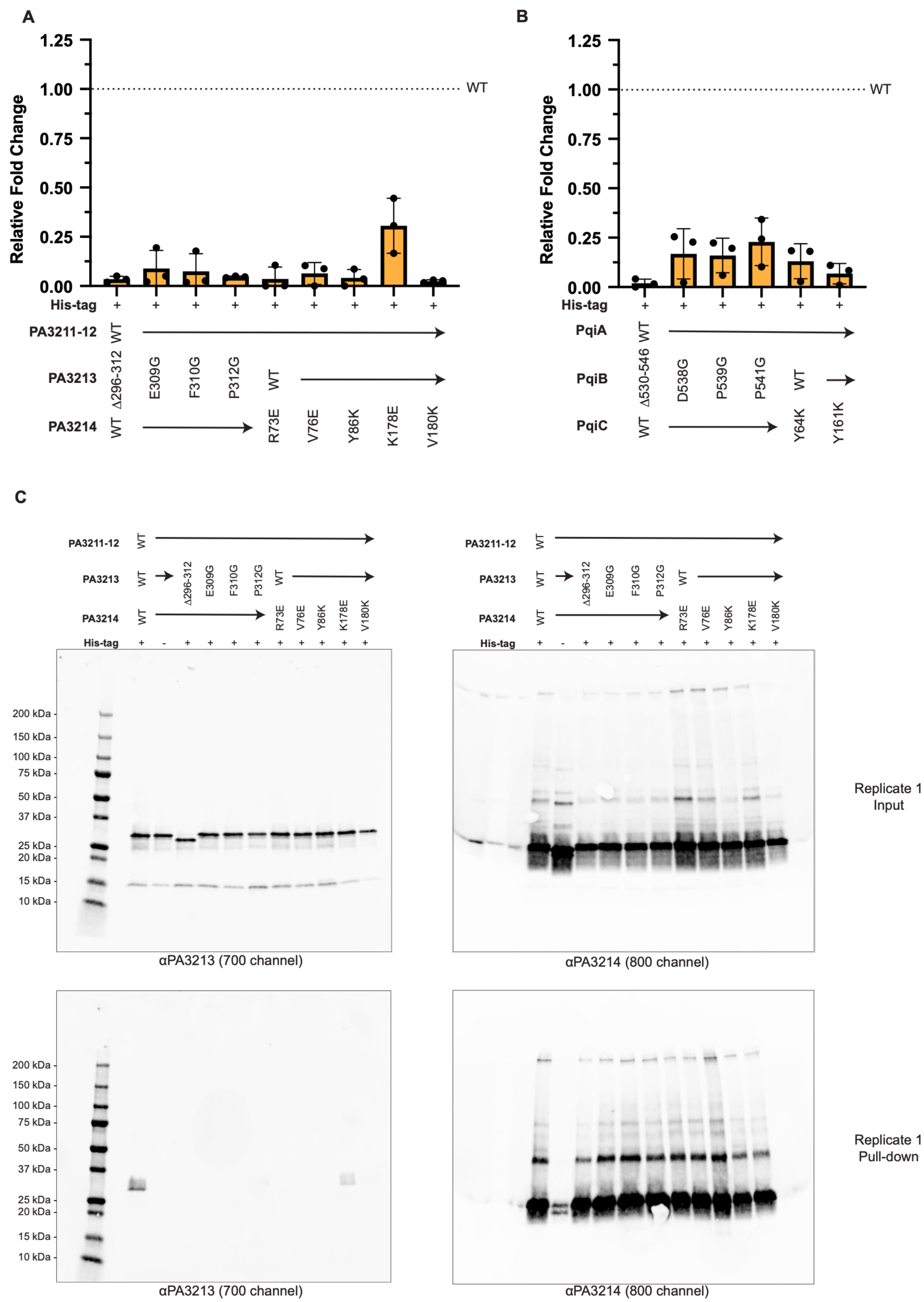
A
B
C
Relative Fold Change
WT
His-tag
PA3211-12
PA3213
PA3214
PqiA
PqiB
PqiC
Δ296-312
E309G
F310G
P312G
R73E
V76E
Y86K
K178E
V180K
Δ530-546
D538G
P539G
P541G
Y64K
Y161K
αPA3213 (700 channel)
αPA3214 (800 channel)
Replicate 1
Input
Replicate 1
Pull-down

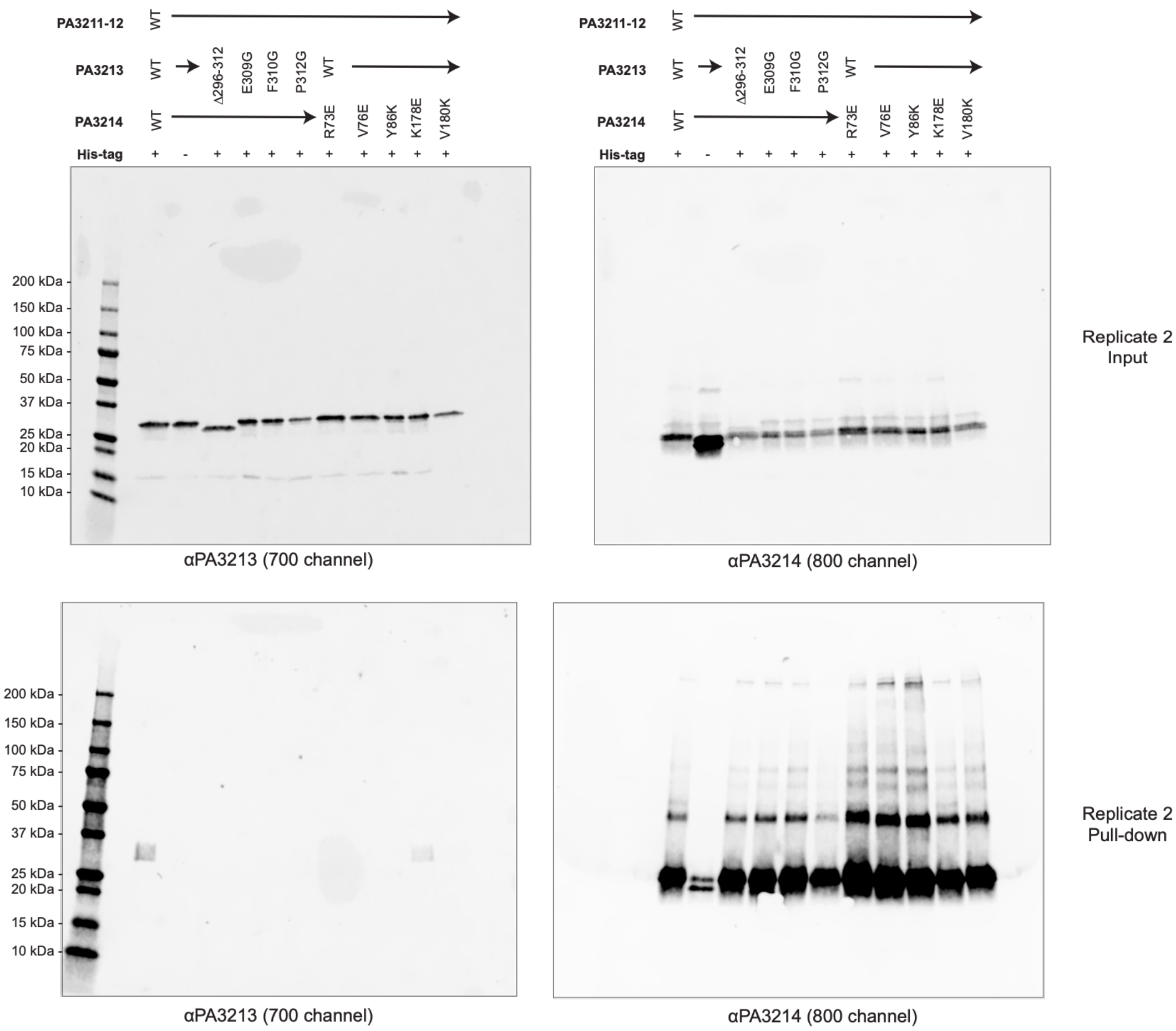
PA3211-12
WT
PA3213
WT
Δ296-312
E309G
F310G
P312G
WT
PA3214
WT
R73E
V76E
Y86K
K178E
V180K
His-tag
+
-
200 kDa
150 kDa
100 kDa
75 kDa
50 kDa
37 kDa
25 kDa
20 kDa
15 kDa
10 kDa
αPA3213 (700 channel)
αPA3214 (800 channel)
Replicate 2
Input
Replicate 2
Pull-down

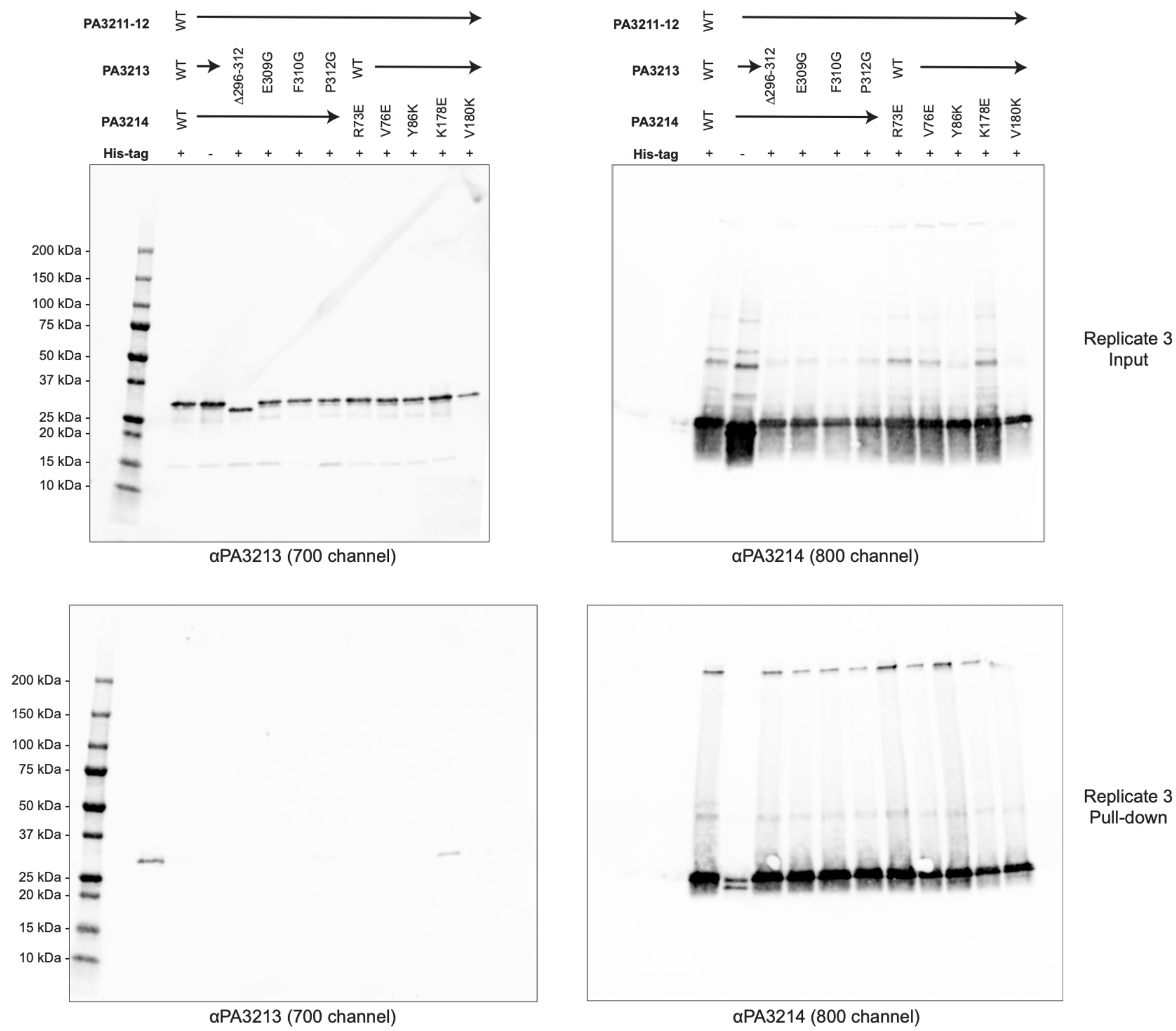

PA3211-12
WT
PA3213
WT
Δ296-312
E309G
F310G
P312G
WT
PA3214
WT
R73E
V76E
Y86K
K178E
V180K
His-tag
+ - + + + + + + + + +
200 kDa
150 kDa
100 kDa
75 kDa
50 kDa
37 kDa
25 kDa
20 kDa
15 kDa
10 kDa
αPA3213 (700 channel)
αPA3214 (800 channel)
Replicate 3
Input
Replicate 3
Pull-down

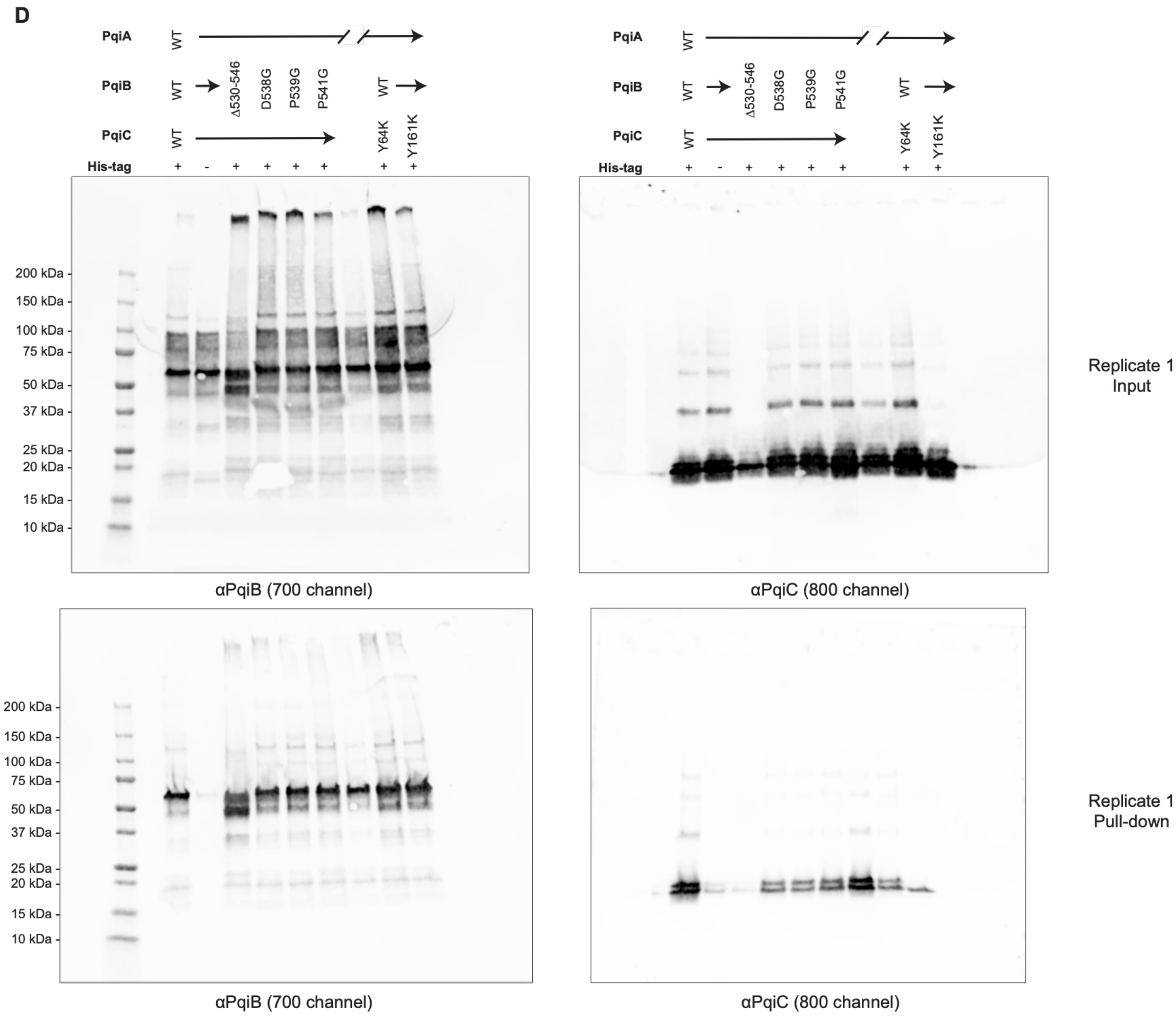
D
PqiA
WT
PqiB
WT
Δ530-546
D538G
P539G
P541G
WT
PqiC
WT
Y64K
Y161K
His-tag
+
-
200 kDa
150 kDa
100 kDa
75 kDa
50 kDa
37 kDa
25 kDa
20 kDa
15 kDa
10 kDa
αPqiB (700 channel)
αPqiC (800 channel)
Replicate 1
Input
Replicate 1
Pull-down

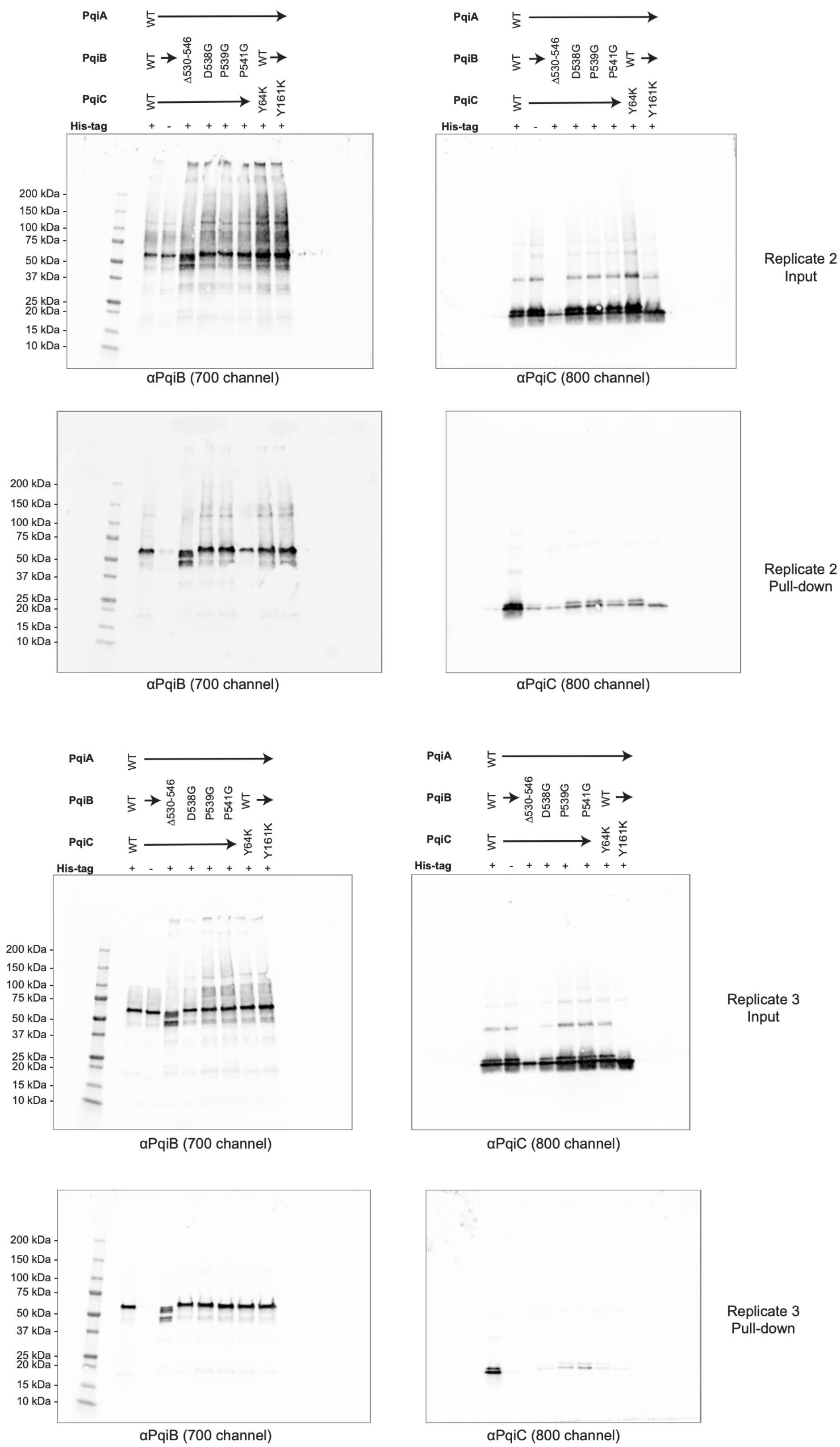
PqiA
WT
PqiB
WT
Δ530-546
D538G
P539G
P541G
WT
PqiC
WT
Y64K
Y161K
His-tag
+ - + + + + + +
200 kDa
150 kDa
100 kDa
75 kDa
50 kDa
37 kDa
25 kDa
20 kDa
15 kDa
10 kDa
αPqiB (700 channel)
αPqiC (800 channel)
Replicate 2
Input
Replicate 2
Pull-down
Replicate 3
Input
Replicate 3
Pull-down

Figure S10. Pull down assays probing the interaction between PA3213:PA3214 and PqiB:PqiC. *A*, Quantification of the western blots showing fold change in the pull down of PA3213 mutants by PA3214, relative to WT (dotted line). For gel source data, see *C*. Plotted data are the mean of three biological replicates ($n = 3$) and error bars representing standard deviations are shown. *B*, Quantification of the western blots showing fold change in the pull down of PqiC mutants by PqiB, relative to WT (dotted line). For gel source data, see *D*. Plotted data are the mean of three biological replicates ($n = 3$) and error bars representing standard deviations are shown. *C,* Western blots from pull-down assays to assess the interaction between PA3213 and PA3214. All four subunits of the PA3211-PA3214 complex were over-expressed, with a His tag on the PA3214 bait, and interaction with untagged PA3213 was assessed using an α-PA3213 antibody. A PA3211-PA3214 no tag construct was included as a control for the inherent stickiness of PA3214 to the NiNTA resin. Blots showing the detergent-solubilized membrane fraction of each strain (input, expression control), and the results of the pull-down are shown for three replicates. *D,* Western blots from pull-down assays to assess the interaction between PqiB and PqiC. All three subunits of the PqiABC complex were over-expressed, with a His tag on the PqiB bait, and interaction with untagged PqiC was assessed using an α-PqiC antibody. A PqiABC no tag construct was included as a control for the inherent stickiness of PqiB to the NiNTA resin. Blots showing the solubilized membrane fraction of each strain (input, expression control), and the results of the pull-down are shown for three replicates.

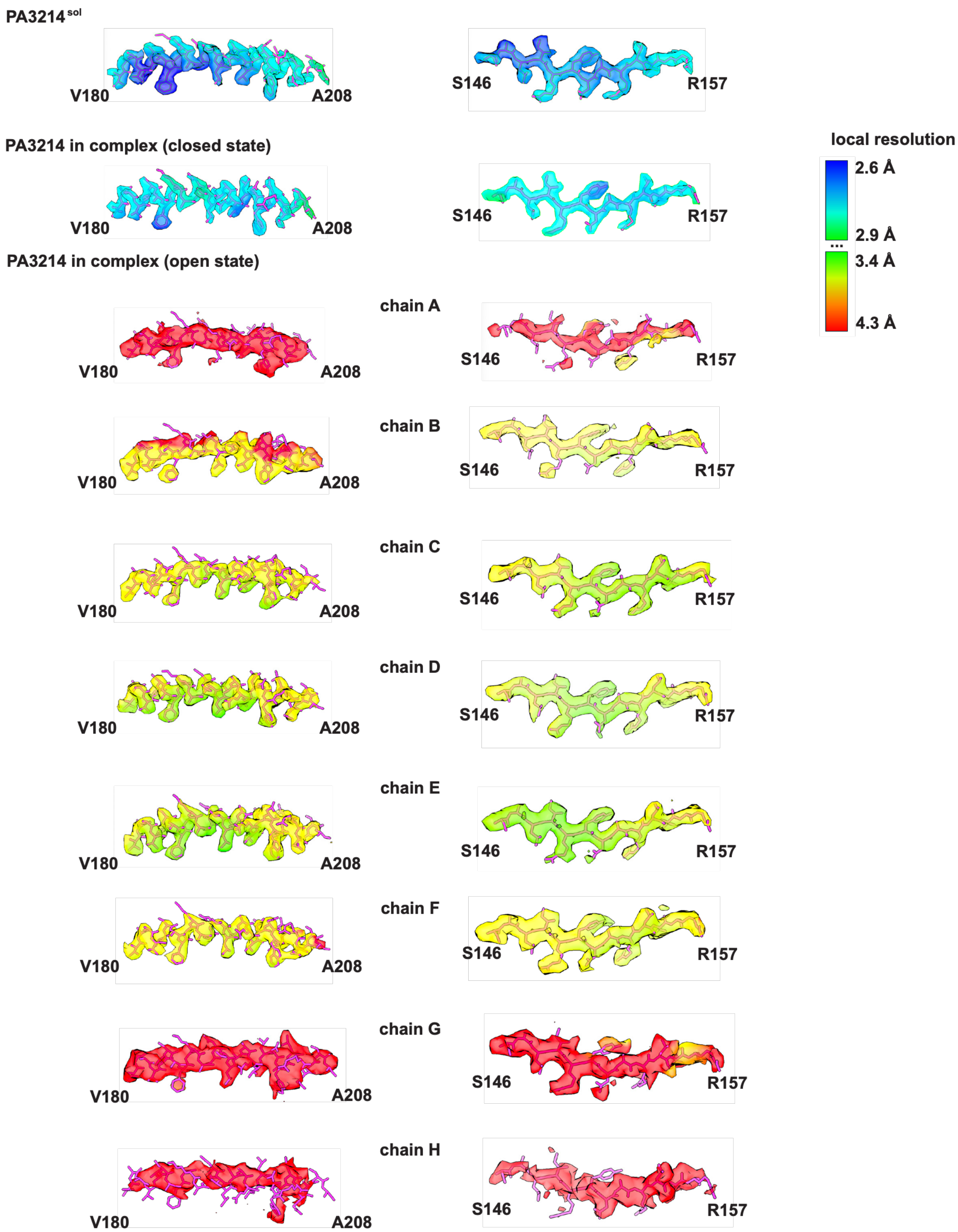

Figure S11. Representative cryo-EM map densities. Model to map fits and representative density from a helix (V180-A208) and a beta-strand (S146-R157) from the different PA3214 structures. All maps are colored by local resolution. Map 1 of PA3214^{sol} is shown sharpened, with C8 symmetry, and at thresholds of 0.768 (S146-R157) and 0.872 (V180-A208); Map 2 of PA3214 from the PA3213-PA3214 subcomplex and in the closed conformation is shown sharpened, with

C8 symmetry, and at thresholds of 1.78 (S146-R157) and 2.22 (V180-A208); Map 3 of PA3214 from the PA3213-PA3214 subcomplex and in the open conformation is shown unfiltered, and at thresholds of 0.541 (S146-R157) and 0.468 (V180-A208).

Supplementary Table S1. cryo-EM data collection for PA3214^{sol}

**Data collection**	
Microscope	Krios1 (PNCC)
Voltage	300
Energy filter	None
Nominal defocus range (µm)	0.8-2.1
Camera	Gatan K3
Detector mode	super resolution
Nominal pixel size (Å/pixel)	0.514425
Nominal magnification	22500x
Dose rate (e-/Å^2 per frame)	50
Frame rate (ms)	48
Exposure time (s)	2.4
Number of movie frames (no.)	50
Automation software	SerialEM
No, of micrographs at 0° tilt	6,463
EMPIAR ID	EMPIAR-13572

Supplementary Table S2. Refinement and validation statistics for PA3214^{sol}

**Data processing**	**Map 1**
Number of particles	654,956
Resolution (Å, FSC=0.143)	2.67
Map sharpening B-factors (Å^2)	-156.2
cFAR	0.73
SCF	0.815
Symmetry	C8
Box size (px)	150
**Model refinement**	
Initial model	homology model using Phyre2
Model composition	
chains	8
non-hydrogen atoms	10784
protein residues	1384
ADP mean B-factors	133.29
R.M.S. deviations	
rmsd (bonds)	0.002
rmsd (angles)	0.564
Molprobity score	1.25
Clashcore, all atoms	4.79
Rotamer outliers (%)	0.00
CaBLAM outliers (%)	1.21
CA Geometry outliers (%)	0.61
FSC (model-map; Å, FSC=0.143/0.5)	2.7 / 2.9
Ramachandran plot (%)	
Favored	98.82
Allowed	1.18
Outliers	0.00
Rama-Z	
whole	-0.79
helix	0.52
sheet	-1.29
loop	-0.54
Map CC (mask)	0.87
Map CC (box)	0.85
Map CC (peaks)	0.73
Map CC (volume)	0.87
EMD ID	EMD-77071
PDB ID	13HQ

Supplementary Table S3. cryo-EM data collection for PA3213-PA3214 subcomplex

**Data collection**	
Microscope	FEI Titan Krios1 (NYSBC)
Voltage	300
Energy filter	Yes
Nominal defocus range (µm)	0.8-3.5
Camera	Gatan K3
Detector mode	counting
Nominal pixel size (Å/pixel)	1.083
Nominal magnification	81000x
Dose rate (e-/Å^2 per frame)	51.51 (untilted) and 51.43 (tilted)
Frame rate (ms)	200
Exposure time (s)	6
Number of movie frames (no.)	30
Automation software	Leginon
No, of micrographs at 0° tilt	6,057
No, of micrographs at 30° tilt	3,925
EMPIAR ID	EMPIAR-13573

Supplementary Table S4. Refinement and validation statistics for PA3213-PA3214 subcomplex

**Data processing**	**PA3214 in closed conformation (Map 2)**	**PA3214 in open conformation (Map 3)**	**Full map (Map 4)**
Number of particles	378,252	187,311	326,143
Resolution (Å, FSC=0.143); (Symmetry)	3.0 (C1)<br>2.7 (C8)	3.7 (C1)	12 (C1)
Map sharpening B-factors ($Å^2$)	-162.7	-181.6	None
cFAR	0.81	0.20	0.22
SCF	0.987	0.964	
Box size (px)	256	256	396
Pixel size (Å)	1.083	1.083	1.444
**Model refinement**			
Initial model	Alphafold & Modelangelo	closed conformation	
Model composition			
chains	16	8	
non-hydrogen atoms	11520	11040	
protein residues	1480	1424	
ADP mean B-factors	141.46	222.46	
R.M.S. deviations			
rmsd (bonds)	0.003	0.004	
rmsd (angles)	0.593	0.669	
Molprobity score	0.91	1.05	
Clashcore, all atoms	1.35	2.68	
Rotamer outliers (%)	0.00	0.00	
CaBLAM outliers (%)	1.69	1.72	
CA Geometry outliers (%)	1.13	0.93	
FSC (model-map; Å, FSC=0.143/0.5)	2.7 / 2.9	3.6 / 3.9	
Ramachandran plot (%)			
Favored	97.79	98.01	
Allowed	2.21	1.99	
Outliers	0.00	0.00	
Rama-Z			
whole	-0.37	-0.73	
helix	0.27	-0.60	
sheet	0.06	0.20	
loop	-0.67	-0.72	
Map CC (mask)	0.89	0.80	
Map CC (box)	0.88	0.86	
Map CC (peaks)	0.78	0.68	
Map CC (volume)	0.89	0.80	
EMD ID	EMD-77072	EMD-77073	EMD-77074
PDB ID	13HR	13HS	

Supplementary Table 5. Bacterial strains and constructs used in this study

Reagent/ Resource	Description	Reference	Addgene ID
Rosetta 2(DE3)	*E. coli* competent cells for protein expression (#714003)	Novagen	
TOP10	*E. coli* competent cells for plasmid propagation (#C404010)	ThermoFisher	
BW25113	*E. coli* K-12 parent strain		
bBEL455	*E. coli* K-12 BW25113 *ΔpqiC*		
pBEL1163	[6xHis-TEV-PqiB(39-546)] expression vector	This study	253832
pBEL1282	PqiA-[6xHis2xQH-TEV-PqiB]-PqiC expression vector	This study	253833
pBEL1296	[PelB-6xHis-TEV-PqiC(17-187)] expression vector	This study	253834
pBEL2277	[6xHis2xQH-TEV-PA3214(27-214)] expression vector	This study	253835
pBEL3076	[6xHis2xQH-TEV-PA3211]-PA3212-PA3213-PA3214 expression vector	This study	253836
pBEL3081	PA3211-PA3212-PA3213-[PA3214-2xHQ6xHis] expression vector	This study	253837
pBEL3085	pET17b-pqiC for complementation	This study	253838
pBEL3438	PqiA-PqiB-PqiC expression vector	This study	253839
pBEL3439	PqiA-[6xHis2xQH-TEV-PqiB(1-530)]-PqiC expression vector	This study	253840
pBEL3440	PqiA-[6xHis2xQH-TEV-PqiB(D538G)]-PqiC expression vector	This study	253841
pBEL3441	PqiA-[6xHis2xQH-TEV-PqiB(P539G)]-PqiC expression vector	This study	253842
pBEL3442	PqiA-[6xHis2xQH-TEV-PqiB(P541G)]-PqiC expression vector	This study	253843
pBEL3444	PqiA-[6xHis2xQH-TEV-PqiB]-PqiC(Y64K) expression vector	This study	253844
pBEL3445	PqiA-[6xHis2xQH-TEV-PqiB]-PqiC(Y161K) expression vector	This study	253845
pBEL3446	PA3211-PA3212-PA3213-PA3214 expression vector	This study	253846
pBEL3447	PA3211-PA3212-PA3213(1-295)-[PA3214-6xHis2xQH] expression vector	This study	253847
pBEL3448	[6xHis2xQH-TEV-PA3214(55-214)] expression vector	This study	253848
pBEL3449	PA3211-PA3212-PA3213(E309G)-[PA3214-6xHis2xQH] expression vector	This study	253849
pBEL3450	PA3211-PA3212-PA3213(F310G)-[PA3214-6xHis2xQH] expression vector	This study	253850
pBEL3451	PA3211-PA3212-PA3213(P312G)-[PA3214-6xHis2xQH] expression vector	This study	253851
pBEL3452	PA3211-PA3212-PA3213-[PA3214(R73E)-6xHis2xQH] expression vector	This study	253852
pBEL3453	PA3211-PA3212-PA3213-[PA3214(V76K)-6xHis2xQH] expression vector	This study	253853
pBEL3454	PA3211-PA3212-PA3213-[PA3214(Y86K)-6xHis2xQH] expression vector	This study	253854
pBEL3455	PA3211-PA3212-PA3213-[PA3214(K178E)-6xHis2xQH] expression vector	This study	253855

pBEL3456	PA3211-PA3212-PA3213-[PA3214(V180K)-6xHis2xQH] expression vector	This study	253856
